# A proteomic adaptation of small intestinal mucosa in response to dietary protein limitation

**DOI:** 10.1038/srep36888

**Published:** 2016-11-14

**Authors:** Chunfu Qin, Kai Qiu, Wenjuan Sun, Ning Jiao, Xin Zhang, Lianqiang Che, Haiyi Zhao, Hexiao Shen, Jingdong Yin

**Affiliations:** 1 State Key Lab of Animal Nutrition, College of Animal Science and Technology, China Agricultural University, Beijing, 100193, P. R. China; 2 Institute of Animal Nutrition, Sichuan Agricultural University, Ya’an, 625099, P. R. China; 3 Genecreate Biological Engineering Co., Ltd., National Bio-industry Base, Wuhan, 430075, P. R. China

## Abstract

Dietary protein limitation (PL) is not only beneficial to human health but also applied to minimize nitrogen excretion in livestock production. However, the impact of PL on intestinal physiology is largely unknown. In this study, we identified 5275 quantitative proteins using a porcine model in which pigs suffered PL. A total of 202 proteins |log_2_ fold-change| > 1 were taken as differentially expressed proteins and subjected to functional and pathway enrichment analysis to reveal proteomic alterations of the jejunal mucosa. Combining with the results of western blotting analysis, we found that protein/carbohydrate digestion, intestinal mucosal tight junction and cell adhesion molecules, and the immune response to foreign antigens were increased in the jejunal mucosa of the pigs upon PL. In contrast, amino acid transport, innate and auto immunity, as well as cell proliferation and apoptosis were reduced. In addition, the expression of functional proteins that involved in DNA replication, transcription and mRNA splicing as well as translation were altered in the jejunal mucosa in response to PL. Furthermore, PL may reduce amino acid transport and cell proliferation through the depression of mTOR pathway. This study provides new insights into the molecular mechanisms underlying the small intestinal response to PL.

Dietary protein limitation (PL) has been shown to exert multiple roles in extending lifespan^1^, improving chronic kidney disease recovery^2^ and enhancing stress resistance^1^,^3^,^4^, as well as minimizing environmental pollution arisen from livestock farming by reducing nitrogen excretion^5^. Nevertheless, whether protein limitation would exert elevated or even negative impacts on intestinal physiological reaction, immune response and protein metabolism is largely unknown.

The small intestine is a major site of protein digestion and amino acid (AA) absorption and metabolism^6^. Before entering the portal vein, intestinal luminal free AA were metabolized in the enterocytes, which significantly modified absorbed AA profile comparing to dietary AA profile^6^,^7^,^8^. Simultaneously, the small intestine mucosa is also an interactive membrane that allows dietary nitrogen compounds to influence intestinal structure, epithelium renewal, cellular metabolism, mucosal immunity, and genetic modification^9^,^10^, which has recently attracted great interest in the field. Thus, further study is required to reveal the proteomic adaptation of the intestinal mucosa membrane in response to PL.

In pigs, growing evidence showed that pigs offered protein-limited diets cannot synthesize sufficient amount of all non-essential amino acids (NEAA) to achieve their maximum growth rate and keep healthy^11^. However, the influences of NEAA deficiency on intestinal physiological reaction, immune response and AA extra-/intra- cellular metabolism remain unclear. In addition, intestinal mucosal AA transporters have been shown to be able to trigger cellular responses that result in functional adaptations in growth, immune response, and energy expenditure appropriate to the alteration of dietary protein supply^12^. Nevertheless, whether PL decreases the expression of AA transporters has been inconsistently shown in previous studies^13^,^14^.

Therefore, in the present study, we employed a porcine model, an excellent animal model for studying human nutrition and digestion physiology^15^,^16^, to investigate the impact of PL on intestinal mucosal proteomics and key physiological functions using the isobaric tags for relative and absolute quantitation (iTRAQ) technique. At the same time, this study is the first report to show high-throughput quantitative proteomic changes in porcine jejunum in response to PL.

## Results

### Animal performance

**Figure 1:**
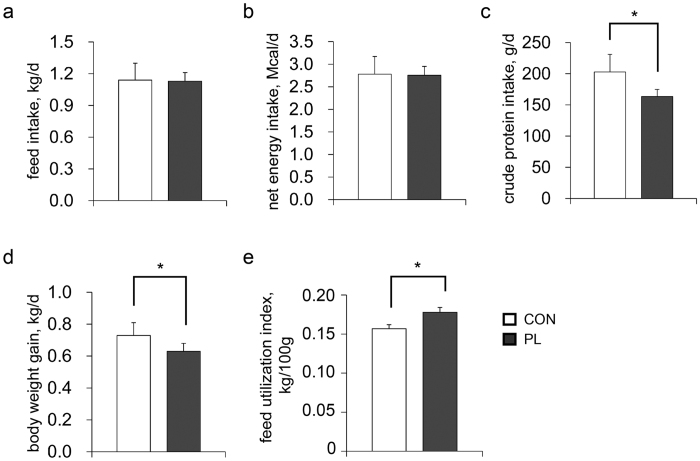
Performance of pigs upon dietary protein limitation. Feed utilization index was defined as feed intake per 100 g of daily body weight gain. *Significant differences between groups; *p* < 0.05. n = 8. CON, control group; PL, dietary protein limitation.

During 28-d PL, we observed that feed intake and net energy intake remained unchanged (*p* > 0.05), whereas protein intake and body weight gain were decreased (*p* < 0.05). The feed utilization index was significantly increased in response to PL (*p* < 0.05) (Fig. 1).

### Small intestinal proteomic profile

In the current study, we analysed three technical replicates from each sample pools of the control (CON) or PL treatment, respectively, with each sample pool consisting of equiponderant proteins isolated individually from the jejunal mucosae of eight pigs. After quality controls (Supplementary file Figure S1), a total of 5275 quantitative proteins were identified based on 65535 spectra from the jejunal mucosae, and 202 proteins with |log_2_ fold-change| > 1 between PL and the CON samples were taken as differentially expressed proteins (DEP), with 178 proteins being up-regulated and 24 being down-regulated upon PL. Details of the proteomics are listed in Supplementary Data.

**Figure 2:**
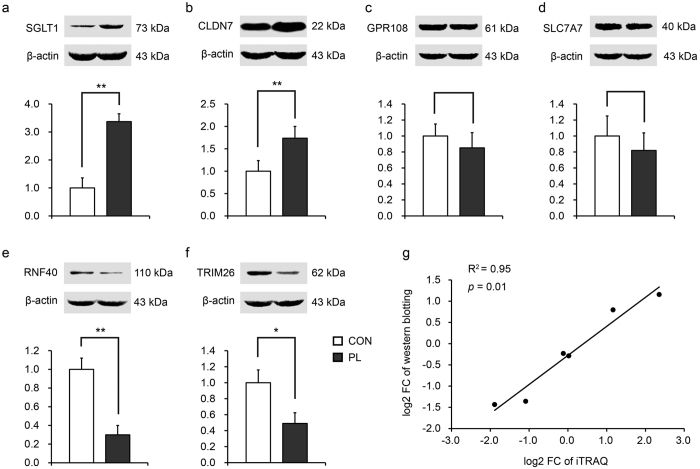
Validation of iTRAQ data by western blotting with beta-actin as a loading control. *Significant differences between groups; *p < 0.05, **p < 0.01. n = 8. CON, control group; PL, dietary protein limitation; FC, fold-change.

To validate the results of iTRAQ analysis, western blotting was employed to determine the relative abundances of six proteins, including sodium/glucose cotransporter 1 (SGLT1, UniProtKB accession no. F1RLV1_PIG), claudin 7 (CLDN7, UniProtKB accession no. C3VPJ4_PIG), G protein-coupled receptor 108 (GPR108, UniProtKB accession no. F1SCN1_PIG), solute carrier family 7 member 7 (SLC7A7, UniProtKB accession no. A8HG48_PIG), ring finger protein 40 (RNF40, UniProtKB accession no. F1RG77_PIG) and tripartite motif-containing protein 26 (TRIM26, UniProtKB accession no. F1RZ52_PIG). Correlation analysis showed a high consistency between the iTRAQ data and the western blotting data (R^2^ = 0.95, *p* = 0.01) (Fig. 2), indicating high reliability of our proteomic workflow.

### Gene ontology (GO) and Kyoto Encyclopedia of Genes and Genomes (KEGG) enrichment analysis

GO and KEGG enrichment analysis were performed with 202 DEP. In GO analysis, we significantly enriched 307 biological processes terms, 62 molecular functions terms, 56 cellular components terms, respectively (*p* < 0.05). Moreover, by matching DEP to the KEGG and NCBI BLAST databases, we enriched 35 KEGG pathways (*p* < 0.05) (Supplementary Data). Based on GO function annotation and KEGG pathway analysis, combining with NCBI and UniProtKB and existing literatures, DEP were classified into five function groups: 1) nutrient digestion, absorption, and metabolism (30.2%, 61 out of 202 DEP); 2) immune function (18.3%, 37 out of 202 DEP); 3) cell renewal (including cell proliferation and apoptosis) (12.4%, 25 out of 202 DEP); 4) regulation of DNA replication and gene expression (17.3%, 35 out of 202 DEP); and 5) others and/or unknown (32.2%, 65 out of 202 DEP). Some DEP participate in multiple biological functions. Table 1 shows the relevant DEP involved in the above key intestinal physiological functions.

### Network of small intestinal physiological pathways upon PL

**Figure 3:**
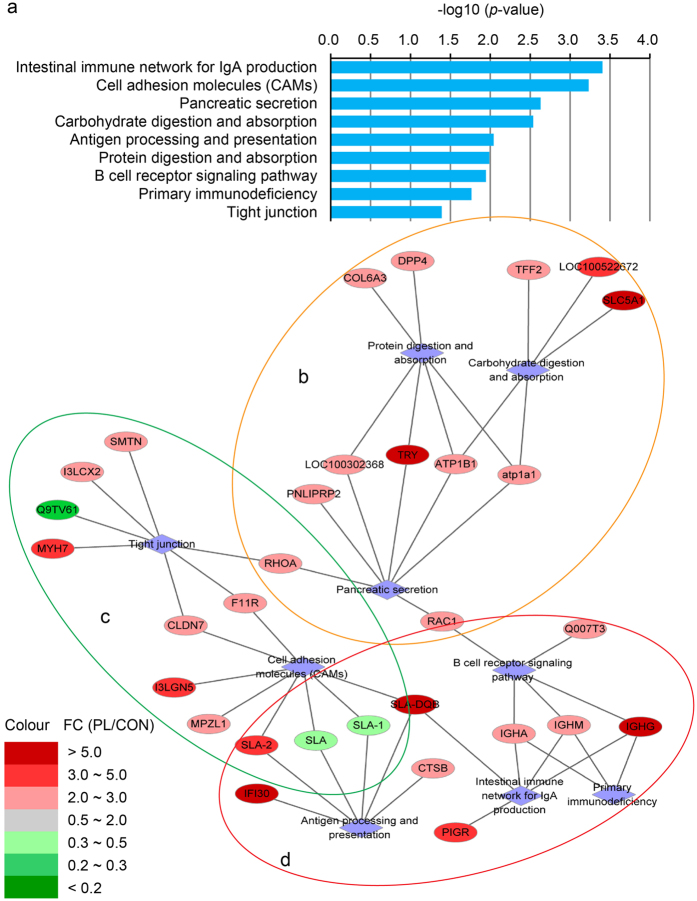
Cluster of KEGG pathways and DEP putatively related to intestinal structure and micro-milieu, protein/carbohydrate digestion and absorption, and immunity in the jejunal mucosa. (**a**) KEGG pathways, the horizontal line represents -log_10_ (*p*-value) for KEGG pathways; DEP putatively related to (**b**) protein digestion and absorption, (**c**) intestinal structure integrity, and (**d**) immunity. (**b**–**d**) Networks were visualized by Cytoscape (3.2.0). DEP, differentially expressed proteins; FC, fold-change; PL, dietary protein limitation; CON, control group.

To obtain an overview on proteomic adaptation of the jejunal mucosa in response to PL, we functionally integrated pathways which are known to be typical intestinal physiology-related pathways via the DEP that are involved among nutrient digestion and absorption, intestinal mucosa epithelium structure and micro-milieu, and intestinal immunity (Fig. 3). We observed that ras homolog family member A (RHOA, UniProtKB accession no. I3LVS7_PIG) that acted as a hub to correlate pancreatic secretion with tight junction was significantly up-regulated upon PL. The up-regulation of MHC class I antigen 2 (SLA-2, UniProtKB accession no. V9PRE5_PIG) and MHC II class antigen (SLA-DQB, UniProtKB accession no. F8J302_PIG) accompanied by the down-regulation of SLA (UniProtKB accession no. Q0MRZ8_PIG) and SLA-1 (UniProtKB accession no. Q8SPB9_PIG) linked the cell adhesion molecules with the antigen processing and presentation in response to PL. Simultaneously, the pathway of pancreatic secretion was associated with B cell receptor signaling pathway through the up-regulated ras-related C3 botulinum toxin substrate 1 (RAC1, UniProtKB accession no. I3LFI0_PIG).

### Protein digestion and amino acid transport

**Figure 4:**
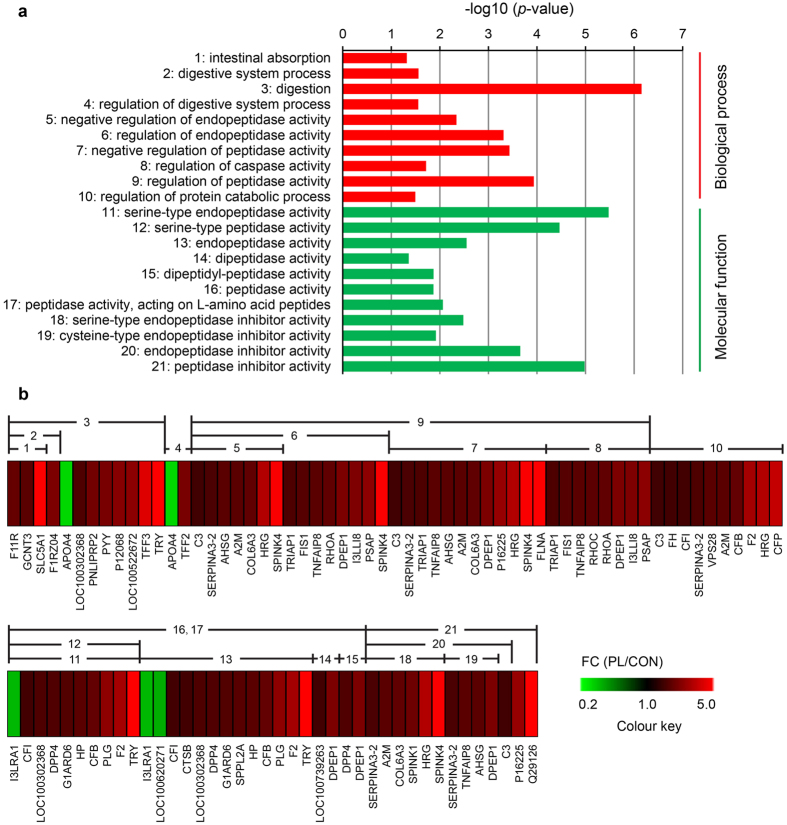
Cluster of GO annotations and DEP putatively related to protein digestion and absorption in the jejunal mucosa. (**a**) The horizontal line represents -log_10_ (*p*-value) for GO terms. (**b**) DEP in each GO terms. Heat-maps were generated by MeV (4.9.0). GO, gene ontology; DEP, differentially expressed proteins; FC, fold-change; PL, dietary protein limitation; CON, control group.

From the significantly enriched GO terms, we clustered 10 biological processes terms, 11 molecular functions terms with regard to protein digestion and transport (Fig. 4), including digestive system process (GO:0022600), regulation of peptidase activity (GO:0052547), peptidase activity (GO:0070011), peptidase inhibitor activity (GO:0030414), intestinal absorption (GO:0050892) and so on.

In the current study, we observed several DEP that were involved in the regulation of intestinal proteolysis and peptide hydrolysis, as well as AA transmembrane transport. For instance, according to UniProtKB, complement factor B (CFB, UniProtKB accession no. F1RQW6_PIG) is an endopeptidase. Both CFB and putative trypsinogen (TRY, UniProtKB accession no. C6L245_PIG) exist in extracellular space and play vital roles in proteolysis. Up-regulation of TRY and CFB implies that proteolysis activity in the intestinal mucosa was increased under PL (Table 1; Supplementary Data). Likewise, the up-regulation of some extracellular peptidases such as dipeptidase 1 (DPEP1, UniProtKB accession no. DPEP1_PIG) implies increased peptide hydrolysis. Thus, we surmised that both proteolysis and peptide hydrolysis activities were increased in the small intestinal lumen in response to PL.

**Figure 5:**
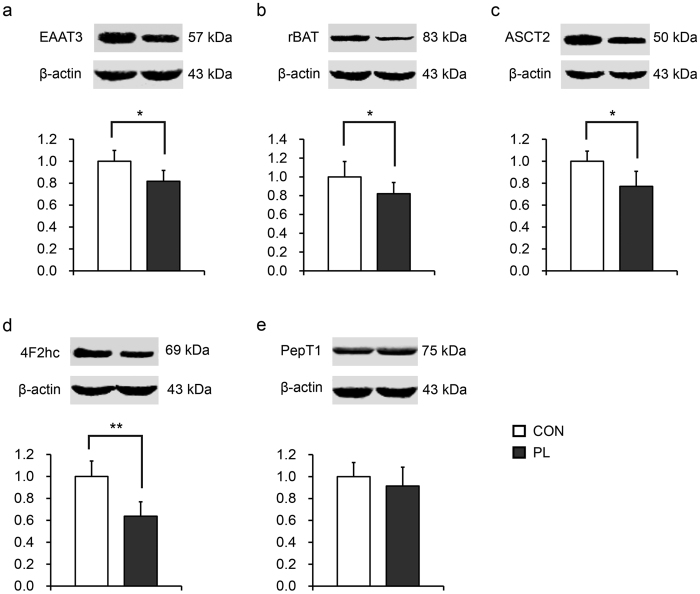
Western blotting analysis of the expression of amino acid/peptide transporters in the jejunal mucosa. *Significant differences between groups; *p < 0.05, **p < 0.01. n = 8. CON, control group; PL, dietary protein limitation.

Results obtained from western blotting analysis indicated that the large neutral AA transporter 4F2 cell-surface antigen heavy chain (4F2hc, UniProtKB accession no. I3LB80_PIG) as well as other AA transporters including excitatory amino acid transporter 3 (EAAT3, UniProtKB accession no. C8CAX4_PIG, an anionic AA transporter), neutral amino acid transporter B(0) (ASCT2, UniProtKB accession no. F1RM08_PIG, a neutral AA transporter), and basic amino acid transport protein rBAT (rBAT, UniProtKB accession no. F1S5K2_PIG, a cationic AA transporter), were down-regulated under PL, whereas peptide transporter-1 (PepT1, UniProtKB accession no. B0ZSN9_PIG) remained unchanged between groups (Fig. 5).

### Protein limitation inhibited phosphorylation of mTOR signalling pathway

**Figure 6:**
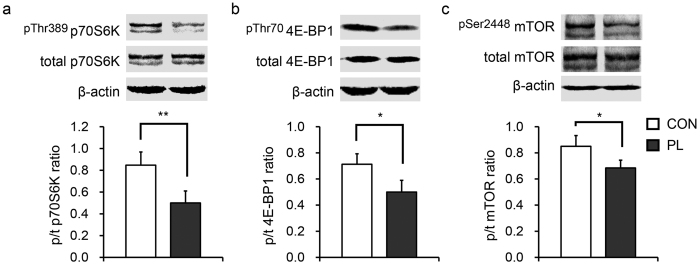
Dietary protein limitation inhibits the activity of mTOR signalling pathway. (**a**–**c**) Dietary protein limitation inhibits phosphorylation of p70S6K and 4E-BP1, as well as mTOR itself. *Significant differences between groups; *p < 0.05, **p < 0.01. n = 8. CON, control group; PL, dietary protein limitation.

Western blotting analysis showed that PL inhibited the phosphorylation of mammalian target of rapamycin (mTOR, Ser2448), p70-S6 kinase (p70S6K, Thr389), and eukaryotic initiation factor 4E binding protein 1 (4E-BP1, Thr70) (Fig. 6).

### Adaptations in intestinal mucosa immunity

We significantly enriched some important GO terms and KEGG pathways such as regulation of humoral immune response (GO:0002920), regulation of inflammatory response (GO:0050727), intestinal immune network for IgA production (ko04672), antigen processing and presentation (ko04612), as well as primary immunodeficiency (ko05340) (Fig. 3). Furthermore, we found that 18.3% of DEP were confirmed to be engaged in immunity, indicating that PL significantly leads to alterations in immune function. As shown in Table 1, SLA-DQB and gamma-interferon-inducible-lysosomal thiol reductase (IFI30, UniProtKB accession no. GILT_PIG) were up-regulated in pigs upon PL. SLA-DQB^17^ and IFI30 had been demonstrated to take part in antigen processing and presentation, moreover, IFI30 plays its role via MHC class I/II^18^. Similarly, IgG heavy chain (IGHG, UniProtKB accession no. L8AXK3_PIG) and poly-Ig receptor (PIGR, UniProtKB accession no. Q9N2H7_PIG) as well as complement factor properdin (CFP, UniProtKB accession no. K7GQR1_PIG) were also up-regulated upon PL. GO annotation showed that complement C1q subcomponent subunit A (C1QA, UniProtKB accession no. C1QA_PIG) and TRIM26^19^ are components of innate immune response that were found among the down-regulated DEP. Histone H4 (HIST4H4, UniProtKB accession no. H4_PIG) constitutes an autoantigen in a H3/4 heterodimer form in auto immunity^20^. NF-kappa B is well-known for its function in immune surveillance, whose inhibitor COMM domain containing 7 (COMMD7, UniProtKB accession no. F1S514_PIG)^21^ was decreased in our study, which suggests a benefit in immunity under PL. Therefore, for the first time, these results suggest that PL probably increases the immune response to foreign antigens. Moreover, it is likely that innate and auto immunity were depressed upon PL.

### Cell proliferation and apoptosis

Considering that 12.4% of DEP found in the jejunal mucosa were involved in cell proliferation and apoptosis, we surmised that the adaptation of cell proliferation and apoptosis occurred in the small intestinal mucosa in respond to PL. We noted that marker of proliferation Ki-67 (MKI67, UniProtKB accession no. F1RXX7_PIG), a well-known endogenous marker of cell proliferation^22^, was down-regulated under PL. Moreover, fatty acid 2-hydroxylase (FA2H, UniProtKB accession no. F1S436_PIG) which promotes cell proliferation was also down-regulated. In addition, IFI30, which negatively regulates cell proliferation and four and a half LIM domains 1 protein isoform C (fhl1C, UniProtKB accession no. Q9GJV4_PIG), which inhibits the mitotic cell cycle, were both up-regulated under PL. These results illustrated that cell proliferation of the small intestine was retarded.

Several DEP involved in apoptosis were also observed, including DPEP1 and ras homolog family member C (RHOC, UniProtKB accession no. F2Z5K4_PIG). GO annotation reveals that DPEP1 is responsible for the negative regulation of apoptotic process and cell migration, meanwhile, RHOC prevents programmed cell death. Based on the result that DPEP1 and RHOC were up-regulated under PL in this study, we speculated that PL may prevent cell apoptosis.

### Regulation of DNA replication and gene expression

17.3% of DEP were observed to take part in the regulation of DNA replication and gene expression. For instance, according to GO annotation, HIST4H4 regulates nucleosome formation, and rho-associated protein kinase 2 (ROCK2, UniProtKB accession no. ROCK2_PIG) positively regulates duplication of centrosome. Given that both HIST4H4 and ROCK2 were down-regulated under PL in this study (Table 1), it is likely that DNA replication was suppressed upon PL.

Both coiled-coil-helix-coiled-coil-helix domain containing 2 (CHCHD2, UniProtKB accession no. F1RIU9_PIG)^23^ and TRIM26^19^ are transcription activators, however, CHCHD2 was up-regulated whilst TRIM26 was down-regulated under PL. Additionally, tyrosine 3-monooxygenase/tryptophan 5-monooxygenase activation protein theta (YWHAQ, UniProtKB accession no. F1SA98_PIG), a transcriptional inhibitor^24^, was up-regulated under PL. U2 small nuclear RNA auxiliary factor 1-like 4 (U2AF1L4, UniProtKB accession no. F1RM65_PIG), a pre-mRNA splicing promoter, was up-regulated while splicing factor 3b subunit 4 (SF3B4, UniProtKB accession no. F1SDG0_PIG), a positive regulator of mRNA splicing was down-regulated (Table 1) upon PL. The complicated alterations of DEP related with transcription and RNA splicing showed that PL exerts a sophisticated role in regulation of transcription. Particularly, ribosomal protein S5 (RPS5, UniProtKB accession no. F2Z5E6_PIG), which plays an important role in maintaining the accuracy of translation in eukaryotes^25^, was up-regulated, indicating translational fidelity was increased upon PL.

## Discussion

Protein limitation not only contributes to human health in terms of longevity extension and insulin sensitivity enhancing^3^,^26^ but also reduces excessive nitrogen excretion in farm animal production^5^. However, previous studies demonstrated that PL impeded individual growth even though all essential amino acid (EAA) were balanced to fulfil requirements of pigs^14^,^27^, which may be due to NEAA deficiency^28^. Recent studies revealed that supplement of NEAA such as glutamic acid, glycine, and proline to protein-limited diets that contain adequate EAA can restore individual growth in pigs^28^,^29^. The influence of EAA deficiency on individual nutrient metabolism and physiology has been well elucidated. However, the impacts of NEAA deficiency on intestine physiology remain unknown, which may be the primary problem that clinical nutritionists have to face while PL is applied to improve human health and so on. In the present study, to avoid the interferences arisen from gender and sex hormones on the results, we chose healthy castrated male pigs with 44-day of age as the model animals, which were equivalent to humans during the early childhood. We successfully established a PL porcine model, in which pigs consumed significantly less protein compared to those offered the control diet. Considering the equivalent intake of EAA and net energy between the pigs offered the control and LP diet, we believe that any change observed in the current study was due to PL, that is, dietary NEAA limitation.

The small intestine mucosa provides a dynamic and interactive surface between lumen and the internal milieu of the organism. Therefore, it would embody meaningful sense while proteomic profile of the intestinal mucosa has been altered by PL^30^.

In the current study, a total of 5275 quantitative proteins were identified in the jejunal mucosa, among which, 202 proteins with |log_2_ fold-change| > 1 were identified as DEP from pigs under PL. The data of western blotting analysis validated the high reliability of our proteomic analysis. We found that PL remarkably altered the expression of proteins that are involved in a few key physiological functions of the small intestine. For instance, PL influenced protein/carbohydrate digestion and AA transport, intestinal mucosal immune, and cell proliferation and apoptosis, as well as regulation of DNA replication and gene expression.

Under PL, our data suggest that proteolysis (TRY and CFB) and peptide hydrolysis (DPEP1) activities increased in the gut lumen, which demonstrated that protein digestion was enhanced. Furthermore, we also check the protein expression of AA/peptide transporters which reflects the activities of protein absorption. Transporter 4F2hc, located in the basolateral membrane, is a high-affinity bidirectional transporter of large neutral AA such as phenylalanine, tyrosine, leucine, arginine and tryptophan after binding to the light chain AA transporters LAT1/2^12^,^31^. In the present study, the protein expression of 4F2hc as well as other AA transporters including EAAT3, ASCT2, and rBAT, was down-regulated upon PL, which was consistent with previous study^14^, suggesting that PL reduced AA transport from the intestinal lumen into enterocytes through the brush-border membrane and further into blood. It has been documented that a protein-limited diet reduces plasma AA concentration^32^. Therefore, we deduce that increased protein digestion could not remedy the reduction of AA absorption upon PL.

So far, the exact mechanism through which ingested dietary protein mediates small intestinal AA transport remains unclear. Two evolutionarily conserved AA sensing-signalling pathways in mammalian cells are the mTOR and general control non-derepressible (GCN) pathways^12^. mTOR complex 1 (mTORC1) is a central cell growth controller that integrates a wide array of extracellular and intracellular signals to regulate cell growth^33^,^34^. mTORC1 mediates transcription and protein translation, as well as cell growth and proliferation in response to nutrient and growth factor availability, and it does this through the phosphorylation of the key translation regulators p70S6K and 4E-BP1^33^,^35^. The GCN pathway primarily senses intracellular AA availability and is activated when one or more AA are scarce^12^,^36^. However, it has been shown in mice that, unlike the mTOR signalling pathway, GCN2 was dispensable for PL-induced effects^3^. It has been demonstrated that mTOR play a central role in mediating AA sensing, AA transport, cell growth and proliferation, apoptosis and autophagy, as well as immunity^37^. Hence we examined the activity of mTOR signalling pathway to understand its role in proteomic adaptation of the intestinal mucosa upon PL. In the current study, our findings indicated that PL inhibits the activity of mTOR signalling pathway in the jejunal mucosa, which was supported by previous studies in rat placenta^38^, mice liver^3^, as well as in porcine liver^39^.

**Figure 7:**
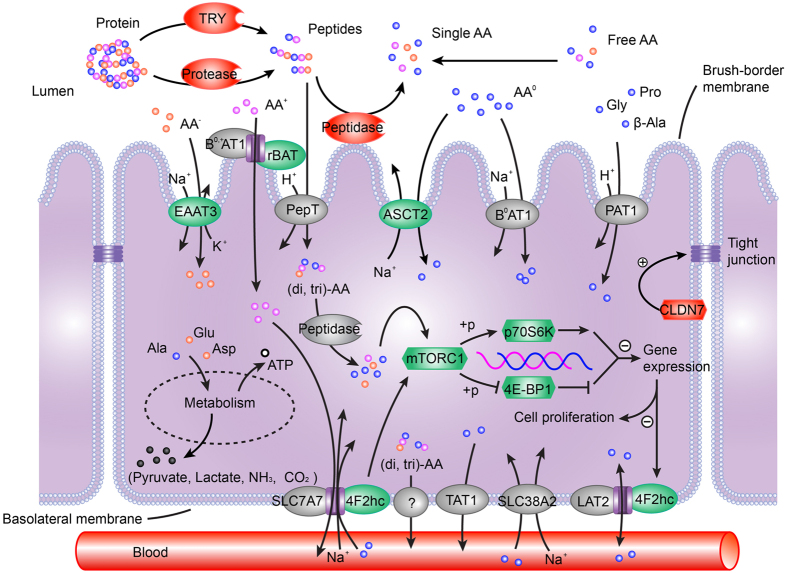
Putative model of mediation of intestinal protein digestion and amino acid transport and mTOR signalling pathway upon protein limitation. Red nodes represent up-regulated proteins, green nodes represent down-regulated proteins upon protein limitation, and grey nodes represent unchanged or undetected proteins in the present study. This model was visualized by Adobe Illustrator (CS5).

Certain AA transporters, 4F2hc and SLC38A2 for instance, may have dual receptor-transporter functions not only act as AA carriers but also as AA sensors^12^,^34^. Thus these AA transporters act directly as the initiating sensors for intracellular AA and mTOR signalling pathway activation^40^. Not only large neutral AA including EAA such as leucine but also certain NEAA such as glutamine are key stimulants of mTOR signalling pathway activation^12^,^41^. Therefore, based on the results of our study, we speculate that PL reduced AA transport and further resulted in extracellular NEAA deficiency, which was sensed by their corresponding AA transporters. This sensing decreased expression of these AA transporters themselves, and thus led to the lack of intracellular NEAA, which subsequently suppressed the mTOR signalling pathway. Finally, the suppressed mTOR signalling pathway would in turn influence a series of biological functions, including the reduction of AA transporter expressions, gene expression as well as intestinal epithelium proliferation and cell growth. A model for mediation of protein digestion and absorption and the activity of mTOR signalling pathway induced by PL is presented in Fig. 7.

Based on the enriched functions involved in cell proliferation and apoptosis, we speculate that PL might inhibit cell proliferation and apoptosis, which had been observed in other cells upon protein restriction^3^,^42^. Moreover, our speculation on cell proliferation can also be underpinned by the inhibited mTOR signalling pathway upon PL in the present study. In addition, CLDN7 plays an important role in controlling intestinal epithelial tight junctions and epithelium differentiation, and its expression increases as epithelial cells differentiate along the intestinal crypte-luminal axis^43^. The tight junction is relevant to extracellular signal perception and the intestinal structure integrity^44^. We observed that CLDN7 expression was increased in the jejunal mucosa, suggesting that tight junctions were enhanced and epithelium differentiation was promoted upon PL.

To the best of our knowledge, very few studies reveal that proteomic alteration correlated with the regulation of DNA replication and RNA transcription in the intestinal mucosa induced by PL. It had been reported that consumption of high-fat diet elevates mouse small intestine mucosa proteins that are involved in transcription, RNA processing, and in the synthesis, modification, and processing of protein^30^. Interestingly, we found that a few DEP have been implicated in DNA replication, transcription and RNA splicing as well as translation fidelity upon PL, even the alterations of DEP that mediate transcription and RNA splicing were complicated, implying that PL has a meaningful but complex effect on intestinal gene expression pattern.

Overall, by employing a porcine model, this study provides the first evidence illustrating the proteomic alteration of the small intestine mucosa in response to PL, suggesting PL exerts profound impacts on small intestine physiology. Our findings indicate that PL increases protein digestion but depresses AA transport. Intestinal innate and auto immune, as well as cell proliferation and apoptosis were mitigated upon PL while the immune response to foreign antigens is enhanced. Our study threw new light on how PL influences intestinal physiological functions, in particular AA transport, intestinal mucosa structure and micro-milieu, as well as intestinal immunity. Thereinto, the mTOR signalling pathway seems to play a central role in mediating intestinal physiological functions upon PL through sensing AA supply.

## Methods

### Feeding trial and tissue harvest

The animal experiment was carried out in accordance with the Chinese Guidelines for Animal Welfare and Experimental Protocol, and was approved by the Animal Care and Use Committee of Sichuan Agricultural University. In this study, 16 44-day old of Duroc × Landrace × Yorkshire crossbred castrated male pigs with an initial body weight of 13.5 ± 0.5 kg were selected from a commercial herd. The pigs were kept individually in the same nursery house for a 3-d adaptation period followed by a 28-d trial period.

Castrated male pigs were randomly assigned by initial body weight to alternative dietary treatments (PL vs. CON; n = 8). Dietary crude protein (nitrogen content × 6.25) content was 18% in CON according to the nutrient recommendation^45^; whilst crude protein content in PL was 14%. Pigs were allowed *ad libitum* access to feed and water. Ingredient composition and nutrient content of the experimental diets are presented in Supplementary file Table S1. Experimental diets were balanced in standardized ileal digestible EAA, net energy, fibre, and electrolytes to meet NRC (2012) nutrient recommendations^45^.

Feed intake and body weight gain were measured individually, while net energy and crude protein intake and the feed utilization index were calculated based on measured data. Net energy or crude protein intake is the dietary net energy or crude protein content multiplied by feed intake, and the feed utilization index was defined as feed intake per 100 g of daily body weight gain. At the end of the feeding trial, castrated male pigs were weighed after 12 h-fasting and then sacrificed by exsanguination post-anaesthesia. Mucosae from the middle jejunal segment were gently scraped. For sampling consistently, an experienced technician harvested the jejunal mucosae throughout the sampling. And then, the mucosal scraps were snap-frozen in liquid nitrogen and stored at −80 °C for further analysis.

Animal performance data (n = 8) were analysed by one-way analysis of variance with each animal as an experimental unit using SAS software (v9.1). Data were expressed as the means and standard error of mean, *p* < 0.05 was considered statistically significant.

### Protein extraction and 8-plex iTRAQ labelling

Individual mucosal scrapings were ground in liquid nitrogen and then lysed in a solution containing 200 μl of L3 buffer (50 mM Tris-Cl, pH 8, 8 M urea, 2 M thiourea, 2 M EDTA, 1× protease inhibitors cocktails), 800 μl of ice-cold acetone, and 10 mM dithiothreitol. The suspensions were incubated at −20 °C for 2 h. After centrifugation at 13,000× *g* for 20 min at 4 °C, the precipitated pellets were resuspended in 800 μl of ice-cold acetone and 10 mM dithiothreitol. The suspensions were further centrifuged at 13,000× *g* for 20 min at 4 °C to collect the precipitated pellets, then vacuum dried. The dried precipitated pellets were dissolved in 200 μl of L3 buffer. Subsequently, total protein concentration was determined using the Bradford assay.

Equiponderant proteins from each animal were pooled based on groups, and then, each pooled sample was divided into three equal parts (named CON1~3 and PL1~3, respectively) as technical triplicates. Subsequently, an aliquot of 100 μg of protein from each fraction was reduced, alkylated, and digested with trypsin according to the manufacturer’s protocol (Applied Biosystems, Framingham, MA, USA). Samples were iTRAQ labelled as following: CON1, 113; CON2, 114; CON3, 115; PL1, 116; PL2, 117; PL3, 118. The labelled peptides were then mixed and vacuum dried.

### 2D-RPLC fractionation and MS/MS analysis

The LC-MS/MS (Triple TOF 5600 plus, AB SCIEX, Framingham, USA) analysis was performed with the modification as described by Ding *et al*.^46^. Briefly, the iTRAQ labelled peptide mixture was reconstituted in reverse phase liquid chromatography (RPLC) mobile phases buffer A (20 mM ammonium formate in 2% acetonitrile, pH 10.0) and subsequently fractionated using a high pH C18 high-performance liquid chromatography system (Dinoex Ultimate 3000 BioRS, Thermo Fisher, Waltham, MA, USA) equipped with Durashell-C18 reverse phase column (5 μm particle size, 100 Å pore size, 4.6-mm diameter × 250-mm length). The fractionation was performed for 50 min at a flow rate of 1 ml/min. Eluted fractions were monitored by measuring the absorbance at a wavelength of 214 nm. Each fraction was desalted with a Strata X C18 column (Phenomenex, Torrance, CA, USA). Samples were vacuum dried and then reconstituted in 15 μl of buffer (0.1% formic acid, 2% acetonitrile in Milli-Q water). Fractions from the above high pH C18 RPLC were further separated by a 90 min linear gradient of 5% to 76.4% acetonitrile in 0.1% formic acid at a flow rate of 350 nl/min. The MS scan range was 350–1250 *m/z* for 0.25 s scanning. Subsequently, MS/MS scans were performed for 0.04 s by applying a spray voltage of 2300 V. The dynamic exclusion for MS/MS was set at 12 s. The MIAPE reporting guidelines are followed throughout this work^47^.

### Protein identification and quantification

Protein identification and quantification for iTRAQ experiments were performed using ProteinPilot (v4.0.8085) software (Supplementary file Table S2). The LC-MS/MS data were searched against the UniProtKB (*sus scrofa*). To minimize the false discovery rate, a threshold for protein identification was applied, with the confident value ≥95% (amount to the confident value “unused ProtScore” ≥1.3 in ProteinPilot software), and at least one unique peptide was considered for protein identification. Proteins that were quantified with |log_2_ fold-change| > 1 were considered to be DEP. The mass spectrometry proteomics data have been deposited to the ProteomeXchange Consortium via the PRIDE partner repository^48^ with the dataset identifier PXD004069.

### GO and KEGG enrichment analysis

We matched our proteomic data to GO and KEGG enrichments by matching Blast2GO software or KEGG database to the NCBI database. Fisher Exact test was used to identify the significantly enriched GO terms or KEGG pathways in the DEP compared to the proteomic background. The formula was the following:


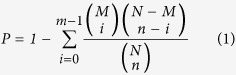


where, N was the number of all identified proteins with GO or KEGG annotation; n was the number of DEP in N; M was the number of all identified proteins that were annotated to the specific GO term or KEGG pathway; m was the number of DEP in M. The *p* < 0.05 were considered as the threshold for significant GO or KEGG enrichment.

### Western blotting

The relative abundances of proteins selected for verification (SGLT1, CLDN7, GPR108, SLC7A7, RNF40, TRIM26), AA transporters (EAAT3, rBAT, ASCT2, 4F2hc), PepT1, as well as mTOR signalling pathway members (mTOR, p-mTOR, p70S6K, p-p70S6K, 4E-BP1, p-4E-BP1) were determined by western blotting analysis using the protein samples from all individual animals (n = 8). Beta-actin was run as a loading control. Equal amounts of samples (80 μg protein per lane), together with a pre-stained protein ladder (Thermo Fisher, Rockford, IL, USA), were subjected to SDS-PAGE electrophoresis, subsequently, proteins were electrotransfered onto a polyvinylidene difluoride membrane (Millipore, Bedford, MA, USA) and then blocked in 5% (w/v) bovine serum albumin at room temperature for 1 h. The membranes were then incubated against corresponding primary antibodies (Supplementary file Table S3) at room temperature for 1 h and then at 4 °C overnight. After three washes, the membranes were incubated with DyLight 800-labeled secondary antibodies (KPL, Gaithersburg, MD, USA). Finally, band intensities were detected with the Odyssey Clx (4647 Superior Street, LI-COR Biotechnology, Lincoln, NE, USA) and quantified using AlphaImager software (2200), then analysed by Mann-Whitney U test using SPSS software (v20). The correlation between the western blotting and iTRAQ data were assessed using PROC CORR procedure of SAS (9.1).

## Additional Information

**How to cite this article**: Qin, C. *et al*. A proteomic adaptation of small intestinal mucosa in response to dietary protein limitation. *Sci. Rep.* **6**, 36888; doi: 10.1038/srep36888 (2016).

**Publisher’s note:** Springer Nature remains neutral with regard to jurisdictional claims in published maps and institutional affiliations.

## Supplementary Material

Supplementary Information

Supplementary Data

## Figures and Tables

**Table 1:** Intestinal key physiological functions and relevant differentially expressed proteins in the jejunal mucosa of pigs upon dietary protein limitation.

UniProtKB accession no.	protein name	associated gene name	FC	function
**Nutrient digestion, absorption, and metabolism**				
up-regulated				
WAP3_PIG	protein WAP-3	WAP-3	7.26	peptidase inhibitor
C6L245_PIG	putative trypsinogen	TRY	5.58	protein digestion and absorption
F1RLV1_PIG	sodium/glucose cotransporter 1	SGLT1	5.11	digestive system process
F1SFI5_PIG	histidine-rich glycoprotein	HRG	3.79	cysteine-type endopeptidase inhibitor
DPEP1_PIG	dipeptidase 1	DPEP1	2.96	extracellular peptidase
down-regulated				
F1RG77_PIG	ring finger protein 40	RNF40	0.27	proteolysis involved in cellular protein catabolic process
F1SKG5_PIG	putative glycerol kinase 5	GK5	0.32	citrate cycle; glycerolipid metabolism
I3LRA1_PIG	lon peptidase 2	LONP2	0.43	serine-type endopeptidase activator (cytoplasm)
**Immunity**				
up-regulated				
F8J302_PIG	MHC class II antigen	SLA-DQB	36.57	immune response
GILT_PIG	gamma-interferon-inducible-lysosomal thiol reductase	IFI30	5.61	antigen processing
L8AXK3_PIG	IgG heavy chain	IGHG	4.57	response to wounding; intestinal immune network for IgA production
K7GQR1_PIG	complement factor properdin	CFP	4.06	humoral immune and inflammatory response
Q9N2H7_PIG	poly-Ig receptor	PIGR	3.92	polymeric immunoglobulin receptor
down-regulated				
F1S514_PIG	COMM domain containing 7	COMMD7	0.30	negative regulation of NF-kappa B transcription factor activity
Q0MRZ8_PIG	MHC class I antigen	SLA	0.41	antigen processing; allograft rejection
C1QA_PIG	complement C1q subcomponent subunit A	C1QA	0.46	complement activation; innate immune response
H4_PIG	histone H4	HIST4H4	0.47	autoantigen component
F1RZ52_PIG	tripartite motif-containing protein 26	TRIM26	0.47	innate immune response; negative regulation of viral entry into host cell
**Cell renewal**				
up-regulated				
GILT_PIG	gamma-interferon-inducible-lysosomal thiol reductase	IFI30	5.61	negative regulation of cell proliferation
F1SFI5_PIG	histidine-rich glycoprotein	HRG	3.79	negative regulation of cell growth and proliferation
Q9GJV4_PIG	four and a half LIM domains 1 protein isoform C	fhl1C	3.66	negative regulation of G1/S and G2/M transition of mitotic cell cycle
DPEP1_PIG	dipeptidase 1	DPEP1	2.96	negative regulation of apoptotic process and cell migration
F2Z5K4_PIG	ras homolog family member C	RHOC	2.47	negative regulation of programmed cell death
down-regulated				
F1RXX7_PIG	marker of proliferation Ki-67	MKI67	0.34	a marker of cellular proliferation
MEA1_PIG	male-enhanced antigen 1	MEA1	0.45	may play an important role in spermatogenesis and/or testis development
F1SGC0_PIG	FYVE, rhoGEF and PH domain containing 4	FGD4	0.47	involved in the actin cytoskeleton and cell shape
F1S436_PIG	fatty acid 2-hydroxylase	FA2H	0.48	regulation of cell proliferation
**Regulation of DNA replication and gene expression**				
up-regulated				
F1RM65_PIG	U2 small nuclear RNA auxiliary factor 1-like 4	U2AF1L4	12.93	pre-mRNA splicing factor
Q53DY7_PIG	histone H1.3-like protein	LOC595122	3.78	nucleosome assembly
F2Z5E6_PIG	ribosomal protein S5	RPS5	3.58	RNA binding; regulation of translational fidelity
F1RIU9_PIG	coiled-coil-helix-coiled-coil-helix domain containing 2	CHCHD2	3.17	positive regulation of transcription from RNA polymerase II promoter
F1SA98_PIG	tyrosine 3-monooxygenase/tryptophan 5-monooxygenase activation protein, theta	YWHAQ	3.06	negative regulation of transcription
down-regulated				
F1SDG0_PIG	splicing factor 3b subunit 4	SF3B4	0.46	positive regulation of mRNA splicing
H4_PIG	histone H4	HIST4H4	0.47	nucleosome formation; chromatin organization
F1RZ52_PIG	tripartite motif-containing protein 26	TRIM26	0.47	positive regulation of sequence-specific DNA binding transcription factor activity
F1SPG1_PIG	histone H1x	H1FX	0.49	nucleosome assembly
ROCK2_PIG	rho-associated protein kinase 2	ROCK2	0.50	positive regulation of centrosome duplication

FC, fold-change calculated by the abundance of a specific protein upon dietary protein limitation divided by it in control pigs.
