# Supplementary material for: A proteomic adaptation of small intestinal mucosa in response to dietary protein limitation
Source: Sci Rep. 2016 Nov 14;6:36888. doi: 10.1038/srep36888 (PMC5107940; doi:10.1038/srep36888)
Supplement: Supplementary Information [file srep36888-s1.pdf]

Supplementary file

A proteomic adaptation of small intestinal mucosa in response to dietary protein limitation

Chunfu Qin[1], Kai Qiu[1], Wenjuan Sun[1], Ning Jiao[1], Xin Zhang[1], Lianqiang Che[2,*], Haiyi Zhao[3], Hexiao Shen[3] & Jingdong Yin[1,*]

[1]State Key Lab of Animal Nutrition, College of Animal Science and Technology, China Agricultural University, Beijing, 100193, P. R. China

[2]Institute of Animal Nutrition, Sichuan Agricultural University, Ya'an, 625099, P. R. China

[3]Genecreate Biological Engineering Co., Ltd., National Bio-industry Base, Wuhan, 430075, China

[*] Correspondence should be addressed to J.Y. (email: yinjd@cau.edu.cn) or L.C. (email: clianqiang@hotmail.com)

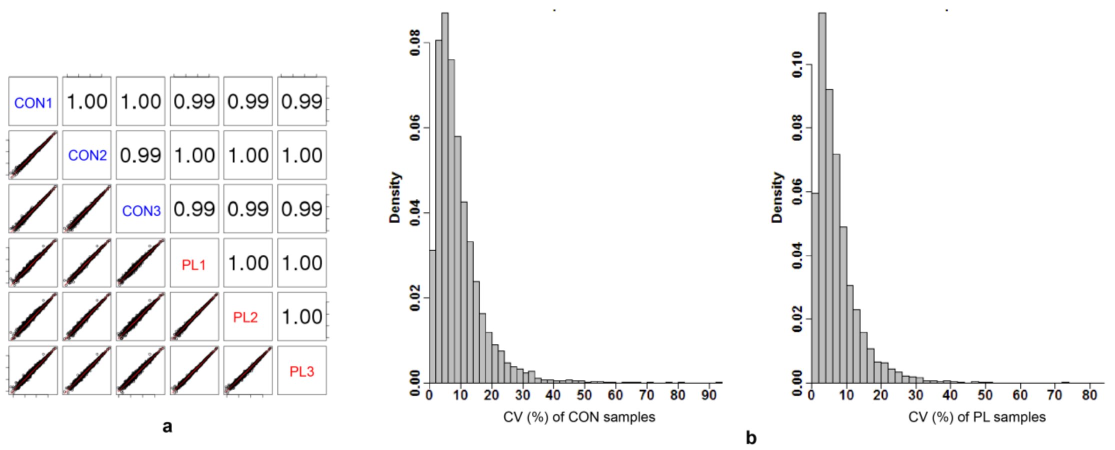

Figure S1. Quality controls of iTRAQ analysis. (a) All correlation coefficients of protein abundance between any of two replicates were above 0.99. (b) CV distributions in both groups were mainly distributed in range from 0 to 20%. This figure indicates high repeatability of the technical replicates. CON, control group. PL, dietary protein limitation. CV, coefficient of variation. n = 3.

Ingredients	CON	PL	Nutrient contents	CON	PL
Corn	67.48	67.44	Net energy, Mcal/kg	2.44	2.44
Soybean meal	10.56	2.49	Crude protein	17.89	14.07
Canola meal	2.17	0.51	Crude fiber	3.48	3.48
Cottonseed meal	2.17	0.51	Lysine	1.23	1.23
Soy protein concentrate	4.00	4.00	Methionine	0.36	0.37
Fish meal	3.00	3.00	Methionine + Cysteine	0.68	0.69
Rice bran	1.14	3.43	Threonine	0.73	0.73
Corn starch	4.24	12.28	Tryptophan	0.20	0.20
L-Lysine HCl	0.57	0.89	Valine	0.78	0.78
DL-Methionine	0.24	0.37	Isoleucine	0.63	0.63
L-Threonine	0.22	0.37	Leucine	1.27	1.23
L-Tryptophan	0.06	0.11	Phenylalanine	0.75	0.73
L-Valine	0.12	0.32	Phenylalanine + Tyrosine	1.20	1.15
L-Isoleucine	0.06	0.25	Histidine	0.43	0.42
L-Leucine		0.27	Arginine	0.94	0.61
L-Phenylalanine		0.31	Calcium	0.70	0.70
L-Histidine HCl		0.13	STTD phosphorus	0.33	0.33
Soybean oil	1.20	0.40			
Limestone	0.83	0.84			
Dicalcium phosphate	0.54	0.68			
Sodium chloride	0.30	0.30			
Choline chloride, 50%	0.10	0.10			
Vitamin-mineral premix	1.00	1.00			

Table S1. Ingredients and nutrient contents of experimental diets (as-fed basis %). CON, control group. PL, dietary protein limitation. Vitamin-mineral premix supplies the following (per kg of feed): vitamin A, 10 KIU; vitamin D_3, 30 IU, vitamin E, 30 IU; vitamin K_3, 6 mg; vitamin B_1, 2 mg; vitamin B_2, 6 mg; vitamin B_6, 3 mg; vitamin B_{12}, 20 μg; niacin, 40 mg; pantothenic acid, 24 mg; folic acid, 1.3 mg; biotin, 80 μg; Fe, 180 mg; Cu, 300 μg; Zn, 200 μg; Mn, 40 mg; I, 960 μg; Se, 720 μg. Amino acid contents are based on standardized ileal digestibility. STTD, standardized total tract digestible.

Parameter	Setting
Type of search	iTRAQ 8-plex (peptide labeled)
Enzyme	Trypsin
Cys Alkylation	Lodoacetic acid
Instrument	TripleTOF 5600
Bias Correction	TRUE
Background Correction	TRUE
ID focus	Biological modifications
Search Effort	Thorough ID
Protein Mass	Unrestricted
Database	UniProt Pig (33958 entries, released October 2014)

Table S2. ProteinPilot parameter settings.

Antibody	Catalog no.	Source	Clone	MW	Dilution	Company	Producing area
SGLT1/SLC5A1	bs-1128R	Rabbit	Poly	73	1: 250	BIOSS	Beijing, China
CLDN7	34-9100	Rabbit	Poly	22	1:1000	Invitrogen	Carlsbad, CA, USA
GPR108	sc-247056	Goat	Poly	61	1: 200	Santa Cruz	Dallas, TX, USA
SLC7A7	sc-34551	Goat	Poly	40	1: 200	Santa Cruz	Dallas, TX, USA
RNF40	bs-9170R	Rabbit	Poly	110	1: 250	BIOSS	Beijing, China
TRIM26	bs-16728R	Rabbit	Poly	62	1: 250	BIOSS	Beijing, China
4F2hc/SCL3A2	bs-6659R	Rabbit	Poly	69	1:500	BIOSS	Beijing, China
ASCT2 /SLC1A5	ARP42247_T100	Rabbit	Poly	60	1:500	AVIVA	San Diego, CA, USA
EAAT3/SLC1A1	SAB2102170	Rabbit	Poly	57	1:1,000	Sigma	St. Louis, MO, USA
rBAT/SCL3A1	sc-32930	Rabbit	Poly	83	1:500	Santa Cruz	Dallas, TX, USA
PepT1/SLC15A1	sc-19917	Goat	Poly	75	1:500	Santa Cruz	Dallas, TX, USA
mTOR	2983	Rabbit	Mono	289	1:1,000	CST	Danvers, MA, USA
Phospho-mTOR (Ser2448)	5536	Rabbit	Mono	289	1:500	CST	Danvers, MA, USA
p70 S6 Kinase	9202	Rabbit	Poly	70, 85	1:1,000	CST	Danvers, MA, USA
Phospho-p70 S6 kinase (Thr389)	9205	Rabbit	Poly	70, 85	1:500	CST	Danvers, MA, USA
4E-BP1	9644	Rabbit	Mono	15-20	1:1,000	CST	Danvers, MA, USA
Phospho-4E-BP1 (Thr70)	13396	Rabbit	Mono	15-20	1:500	CST	Danvers, MA, USA
β-actin	HX1827	Mouse	Mono	43	1:2,000	HXBC	Beijing, China

Table S3. Information of primary antibodies used for western blotting analysis. MW, molecular weight, kDa. BIOSS, Biosynthesis biotechnology; AVIVA, aviva systems biology; Sigma, sigma-aldrich corporation; Santa Cruz, Santa Cruz biotechnology; Invitrogen, invitrogen-thermo fisher scientific; CST, cell signaling technology; HXBC, huaxingbio science.
